# Membrane asymmetry facilitates murine norovirus entry and persistent enteric infection

**DOI:** 10.1371/journal.pbio.3003147

**Published:** 2025-04-17

**Authors:** Brittany M. Stewart, Linley R. Pierce, Mikayla C. Olson, Chengyuan Ji, Robert C. Orchard

**Affiliations:** Departments of Immunology and Microbiology, University of Texas Southwestern Medical Center, Dallas, Texas, United States of America; Duke-NUS Medical School, SINGAPORE

## Abstract

Norovirus, the leading cause of gastroenteritis worldwide, is a non-enveloped virus whose tropism is determined in part by the expression patterns of entry receptors. However, the contribution of cellular lipids to viral entry is not well understood. Here, we determined that the asymmetrical distribution of lipids within membrane bilayers is required for murine norovirus (MNV) replication. Specifically, TMEM30a, an essential subunit of lipid flippases, is required for MNV replication in vitro. Disruption of TMEM30a in mouse intestinal epithelial cells prevents persistent, enteric infection by MNV in vivo. Mechanistically, TMEM30a facilitates MNV binding and entry. Surprisingly, exoplasmic phosphatidylserine (PS), a typical marker of dying cells, does not inhibit MNV infection. Rather, TMEM30a maintains a lipid-ordered state that impacts membrane fluidity that is necessary for the low affinity, high avidity binding of MNV to cells. Our data provides a new role for lipid asymmetry in promoting non-enveloped virus infection in vitro and norovirus persistence in vivo.

## Abstract 2

Norovirus tropism depends partly on entry receptors, but the role of cellular lipids in viral entry remains unclear. This study shows that TMEM30a-mediated lipid asymmetry is crucial for murine norovirus infection, influencing membrane fluidity to support viral binding and entry.

## Introduction

The plasma membrane provides an intrinsic barrier blocking viral access to the host cytosol. Research efforts have largely focused on how receptors enable viruses to cross membranes. In contrast, very little is known about how lipids regulate viral entry. Biological membranes consist of lipid bilayers with asymmetric distribution [1]. Amine-containing phospholipids such as phosphatidylethanolamine (PE) and phosphatidylserine (PS) are found on the inner leaflet of the plasma membrane, but are nearly absent on the outer leaflet. Lipid asymmetry is established and maintained by lipid flippases and floppases. Flippases transport lipids from the exoplasmic side of the membrane to the cytoplasmic side, while floppases catalyze cytoplasmic to exoplasmic transport [2, 3]. It is appreciated that enveloped viruses promote the incorporation of exoplasmic PS in viral envelopes to promote uptake by scavenger receptors [4–6]. This process, coined viral apoptotic mimicry, has been implicated for numerous enveloped viruses including Filoviruses, Orthopoxviruses, and Flaviviruses [5]. However, whether lipid asymmetry is important for non-enveloped virus entry is unknown.

Noroviruses (NoV) are non-enveloped, positive-sense RNA viruses and are a leading cause of gastroenteritis worldwide [7, 8]. Norovirus shedding has been detected in asymptomatic individuals months after symptom resolution [9, 10]. Norovirus persistence has been postulated to be the initiating source of outbreaks, yet our ability to treat or clear persistent norovirus infections is limited [11]. Murine norovirus (MNV) has emerged as the preeminent HNoV model system as it is a natural pathogen of mice, replicates robustly in standard cell lines, and certain MNV strains cause persistent enteric infection of mice that models the long-term shedding of HNoV infections [12–14]. We and others have conducted genome-wide CRISPR screens to identify host genes required for MNV replication [15–17]. In addition to identifying CD300lf as a proteinaceous receptor for MNV, numerous other genes were associated with norovirus replication [15,17]. One such gene, TMEM30a, also called CDC50a, is an essential component of P4-ATPase flippase complexes [18]. Without TMEM30a, lipid flippases are unable to leave the endoplasmic reticulum and cells lose lipid asymmetry; yet how TMEM30a promotes norovirus replication is unclear [19, 20].

Here, we set out to determine how lipid asymmetry maintained by lipid flippases facilitates MNV replication. We determined that TMEM30a is required for replication in vitro and loss of TMEM30a in intestinal epithelial cells prevents persistent, enteric infection by MNV. Interestingly, despite lipid asymmetry being disrupted throughout the cell, MNV only requires TMEM30a for viral entry. In the absence of TMEM30a, MNV is unable to efficiently bind cells even when CD300lf is present on the cell surface. We present a model in which membrane order and fluidity independent of lipid head groups such as PS are important for promoting norovirus infection and persistence.

## Results

### TMEM30a is required for efficient MNV infection in vitro and in vivo

**Fig 1:**
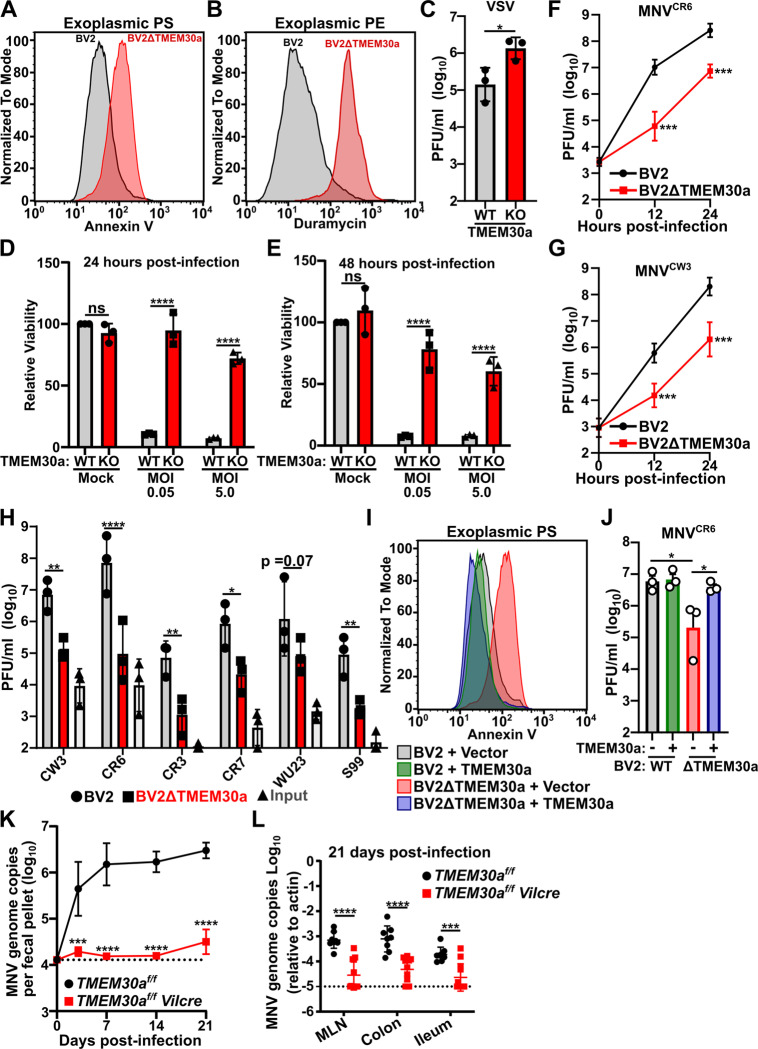
TMEM30a is required for MNV replication in vitro and in vivo. (**A, B)** Representative Histograms comparing Annexin V (**A**; left) or Duramycin (**B**; right) staining of BV2 WT and BV2ΔTMEM30a cells. (**C)** BV2 or BV2ΔTMEM30a cells were challenged with VSV at a multiplicity of infection (MOI) of 0.01. Viral production was enumerated using plaque assays (PFU; plaque forming units) at 16 h post-infection. **(D, E)** BV2 or BV2ΔTMEM30a cells were either mock infected or challenged with MNV^CR6^ at an MOI of 0.05 or 5.0. Twenty-four hours post-infection (**D**; left) or 48 hpost-infection (**E**; right) cellular viability was measured using CellTiter-Glo. All data are normalized to BV2 mock infected. **(F, G)** BV2 or BV2ΔTMEM30a cells were challenged with MNV strains MNV^CR6^ (**F**; top) or MNV^CW3^ (**G**; bottom) at an MOI of 0.05. Viral production was enumerated using plaque assays at the indicated time points. (**H)** BV2 or BV2ΔTMEM30a cells were challenged with indicated MNV strains and 12 h post-infection viral production was enumerated using plaque assay. Viral input is also graphed. (**I)** Representative Histograms comparing Annexin V staining of indicated cell lines. (**J)** BV2 or BV2ΔTMEM30a cells expressing either an empty vector or TMEM30a were challenged with MNV^CR6^ at an MOI of 0.05. Viral production was measured at 12 h post-infection via plaque assay. **(K, L)** *TMEMfl/fl* or *TMEMfl/fl Vilcre* mice were challenged with 10^6^ PFU *per oral* of MNV^CR6^ and genome copies were enumerated in the feces at the indicated times **(K**; left) or in the indicated tissues 21 days post-infection **(L**; right). Each dot represents a single mouse and the dashed lines indicate the limit of detection. All data are shown as mean ± S.D. from three independent experiments and analyzed by one-way ANOVA with Tukey’s multiple comparison test. Statistical significance is annotated as follows: ns not significant, *P < 0.05, **P < 0.01, ***P < 0.001, ****P < 0.0001. The data underlying this Figure can be found in S1 Table.

To verify that TMEM30a is a pro-viral factor for MNV, we generated TMEM30a knockout BV2 cells (BV2ΔTMEM30a) and confirmed that these BV2ΔTMEM30a cells have the anticipated increase in exoplasmic PS and PE via staining with Annexin V and Duramycin respectively (Fig 1A and 1B) [18,21]. Alterations in lipid asymmetry increase enveloped viral spread via apoptotic mimicry, although the specific contribution of TMEM30a to this process is poorly defined [4–6,22]. In line with previous literature on apoptotic mimicry, vesicular stomatitis virus (VSV) showed a modest increase in viral titers in BV2ΔTMEM30a cells compared to wild-type BV2 (Fig 1C) [23]. These data verify known functions of TMEM30a and lipid asymmetry in BV2 cells while also demonstrating that despite global alterations in membrane asymmetry BV2ΔTMEM30a cells can support viral replication. Consistent with this notion, we find no differences in cellular health and viability between BV2 and BV2ΔTMEM30a cells (Fig 1D and 1E). Furthermore, while wild-type BV2 cells succumb to the lytic infection of MNV at both 24 and 48 h post-infection, BV2ΔTMEM30a cells survive viral challenge with both low and high multiplicity of infection (MOI) (Fig 1D and 1E). Taken together these data demonstrate a specific pro-murine norovirus role for TMEM30a that is different from enveloped viral apoptotic mimicry and is not attributed to gross differences in cell health and viability.

MNV strains exhibit diverse cellular and tissue tropisms in vivo [24–26]. We next tested the ability of two representative MNV strains to replicate in BV2ΔTMEM30a cells. MNV^CR6^ causes persistent infection of intestinal tuft cells while MNV^CW3^ infects immune cells causing an acute systemic infection [24,25,27]. Compared to wild-type BV2 cells, BV2ΔTMEM30a cells were deficient in MNV production at a single cycle of replication (12 h) or at multiple cycles (24 h) (Fig 1F and 1G). Similar results were observed with both MNV^CR6^ and MNV^CW3^. We next extended our analysis to a panel of diverse MNV strains that represent major branches on an MNV phylogenetic tree [12]. Twelve hours post-infection BV2ΔTMEM30a cells produced significantly less viral titers compared to wild-type counterparts for MNV strains CW3, CR6, CR3, CR7, and S99 (Fig 1H). We observed a reduction, although not statistically significant, in viral titers for MNV strain WU23 in BV2ΔTMEM30a cells (Fig 1H). Importantly, the defect in viral production for MNV^CR6^ was complemented via expression of a *TMEM30a* cDNA (Fig 1I and 1J). These data indicate that TMEM30a is required for optimal in vitro infection by diverse MNV strains.

Germline deletion of TMEM30a is embryonic lethal [28]. To test the contribution of TMEM30a to MNV infection in vivo, we generated conditional knockout mice (*TMEM*^*fl/fl*^) crossed to Villin Cre (*Vilcre*) to specifically target intestinal epithelial cells, including tuft cells, the reservoir for persistent MNV infections [25,29]. Compared to cre-negative littermate controls, *TMEM30a*^*fl/fl*^*-Vilcre* mice were unable to support persistent fecal shedding of MNV^CR6^ (Fig 1K). Further examination of the tissues demonstrated a dramatic reduction in viral genomes in the colon, ileum, and mesenteric lymph nodes (Fig 1L). These data indicate that TMEM30a is required for MNV to establish a persistent infection in both tissues and fecal shedding.

### TMEM30a is required for viral entry and binding despite CD300lf at the cell surface

**Fig 2:**
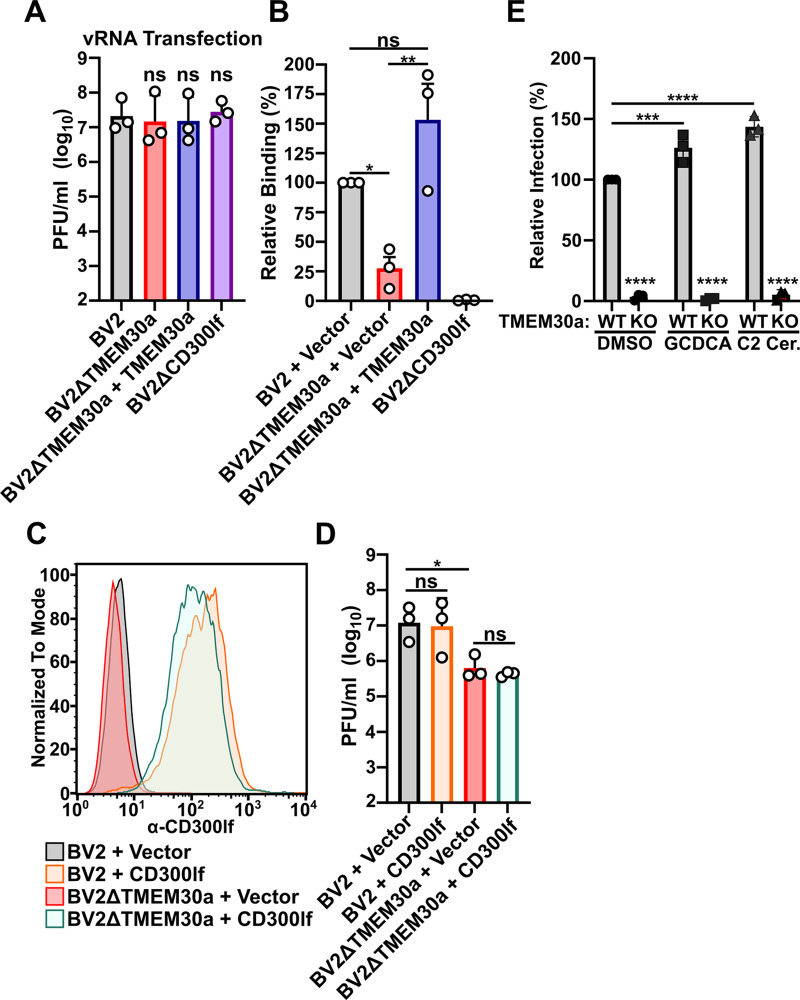
TMEM30a is an MNV entry factor that facilitates viral binding to cells. (**A)** Indicated cell lines were transfected with viral RNA from MNV^CR6^ and harvested 12 h post-transfected. Viral titers were enumerated through plaque assay. (**B)** Indicated cell lines were assayed for MNV^CR6^ binding using quantitative polymerase chain reaction and normalized to the mean of BV2 + vector for each experiment. (**C)** A representative histogram of surface CD300lf staining of indicated cell lines. (**D)** BV2 or BV2ΔTMEM30a cells expressing either an empty vector or CD300lf were challenged with MNV^CR6^ at an MOI of 0.05 and after 12 h post-infection viral production was enumerated via plaque assay. (**E)** BV2 or BV2ΔTMEM30a cells were infected with MNV^CR6^-HiBiT at an MOI 0.5 in the presence of DMSO, 500 µM GCDCA, or 50 µM C2 ceramide. Sixteen hours post-infection cells were lysed and infection was measured using Nano-Glo HiBiT Lytic Detection System (Promega) post-lysis. Samples are normalized to BV2 cells treated with DMSO. All data are shown as mean ± S.D. from three independent experiments and analyzed by one-way ANOVA with Tukey’s multiple comparison test. Statistical significance is annotated as follows: ns not significant, *P < 0.05, **P < 0.01, ***P < 0.001, ****P < 0.0001. The data underlying this Figure can be found in S1 Table.

TMEM30a deficiency impacts lipid asymmetry throughout the cell and has the potential to impact multiple stages of the MNV replication cycle [30]. MNV engages cellular membranes at several points during its replication cycle including the binding of CD300lf at the plasma membrane, establishment of a replication complex via reorganization of the endomembrane system, and viral release from infected cells [31]. Equivalent levels of infectious particles were produced from wild-type BV2 and BV2ΔTMEM30a cells transfected with MNV genomes, indicating that only MNV entry requires TMEM30a (Fig 2A). We then tested the ability of MNV^CR6^ to bind BV2ΔTMEM30a cells. While MNV^CR6^ robustly bound wild-type BV2 cells, it was significantly impaired in binding BV2ΔTMEM30a cells or the viral receptor-deficient BV2ΔCD300lf cells (Fig 2B). Importantly, this binding defect was rescued by expression of TMEM30a cDNA (Fig 2B). Because CD300lf expression is essential for MNV binding and entry, we tested whether overexpression of CD300lf could overcome MNV dependence on TMEM30a for infection. However, there was no improvement in infectious particle production in BV2ΔTMEM30a cells when CD300lf was overexpressed despite the receptor being robustly detected on the surface of the cells (Fig 2C and 2D).

While CD300lf directly interacts with the MNV capsid, it does so through a small surface area with an unusually weak affinity [32]. Therefore, small molecule cofactors have been implicated in enhancing binding affinity and entry for both human and mouse norovirus. Bile acids and ceramides are conserved cofactors that augment norovirus infection in both human and mouse cells [32–34]. For example, the addition of C2 ceramide can rescue the binding defect of MNV in sphingolipid deficient mouse cells while also bypassing the entry restriction of GII.3 human norovirus replication in human intestinal enteroids [33, 34]. Therefore, we tested whether the addition of the bile acid glycochenodeoxycholic acid (GCDCA) or C2 ceramide would rescue MNV replication in BV2ΔTMEM30a cells using a recently developed MNV reporter virus [35]. While addition of GCDCA and C2 ceramide augmented MNV^CR6^ replication in wild-type BV2 cells, the addition of these cofactors did not increase viral replication in BV2ΔTMEM30a cells (Fig 2E). These data indicate that entry requirement for TMEM30a represents an unappreciated step in norovirus entry independent of receptor expression or presence of enhancing cofactors. Taken together, these findings suggest a model in which TMEM30a is required for the entry of MNV while all other steps of viral replication are not significantly affected.

### Membrane order rather than exoplasmic PS is critical for MNV infection

**Fig 3:**
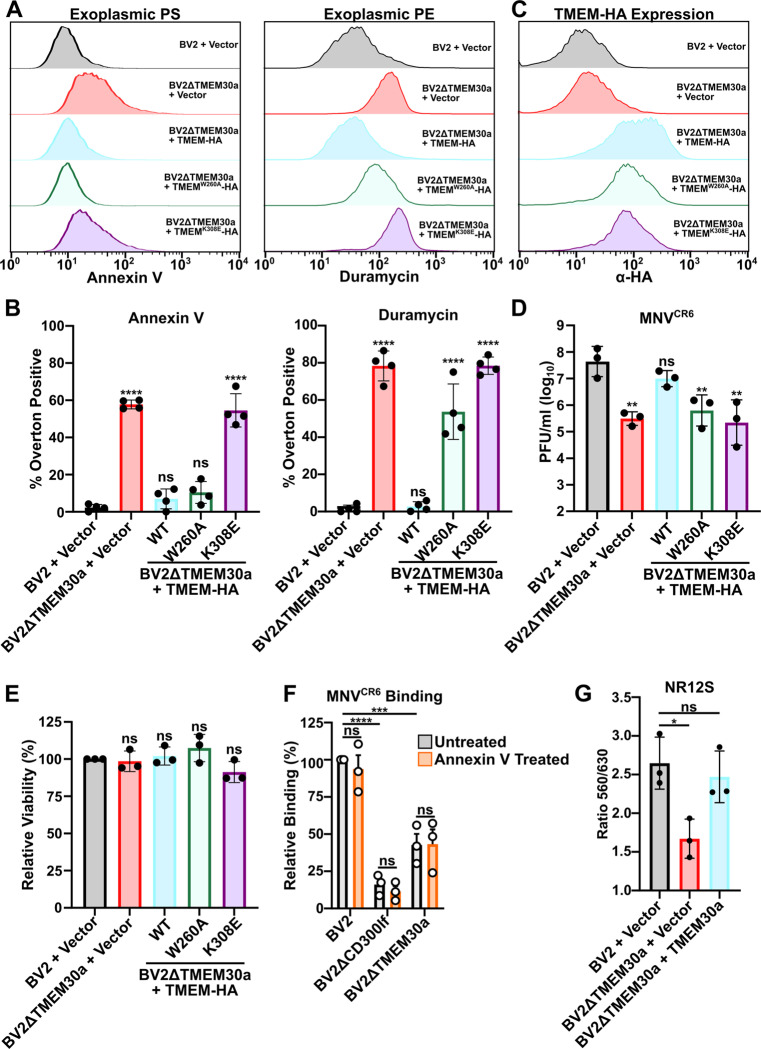
TMEM30a lipid flipping activity of PE but not PS facilitates MNV replication. (**A)** Representative histograms from FACS analysis of indicated cell lines stained with Annexin V (left) or Duramycin (right). (**B)** Quantification of percent positive cells for Annexin V (Left) or Duramycin (right). Data is from four independent experiments. (**C)** Representative histogram of intracellular flow cytometry for TMEM30a-HA of indicated cell lines. (**D)** Indicated cell lines were challenged with MNV^CR6^ at an MOI of 0.05 and viral titers enumerated 12 h post-infection via plaque assay. Data is from three independent experiments. (**E)** Indicated BV2 cell lines were seeded and cellular viability was measured using CellTiter-Glo 24 h later. All data are normalized to BV2 + Vector. (**F)** Indicated cell lines were incubated with either Annexin V or buffer alone on ice prior to performing a binding assay with MNV^CR6^. Within an experiment binding was normalized to BV2 mock treated. Data is from three independent experiments. (**G)** Ratio of the emission at 530 nm over 630 nm of indicated cell lines after incubated with NR12S. Data is from three independent experiments. All data are shown as mean ± S.D. from at least three independent experiments and analyzed by one-way ANOVA with Tukey’s multiple comparison test. Statistical significance is annotated as follows: ns not significant, *P < 0.05, **P < 0.01, ***P < 0.001, ****P < 0.0001. The data underlying this Figure can be found in **S1 Table**.

We next sought to understand how TMEM30a promotes MNV entry. We profiled the exoplasmic PS and PE levels in BV2ΔTMEM30a cells expressing previously reported loss of function mutations to uncouple TMEM30a lipid flipping activities [36]. Fortuitously, we identified cell lines that can be categorized into three distinct plasma membrane states: (1) cells with no exoplasmic PS or PE, (2) cells with both exoplasmic PS and PE, (3) cells with only exoplasmic PE but not exoplasmic PS. More specifically, complementation of BV2ΔTMEM30a cells with TMEM30a reduced exoplasmic PS and PE levels to that of wild-type cells (Fig 3A–3C). In contrast, expression of TMEM30a^K308E^ in BV2ΔTMEM30a cells failed to reduce exoplasmic PS and PE levels (Fig 3A–3C). TMEM30a^K308E^ is unable to chaperone P4-ATPases out of the endoplasmic reticulum [36]. Lastly, expression of the hypomorphic construct TMEM30a^W260A^ led to a discordance in exoplasmic PS and PE levels in BV2ΔTMEM30A cells [36]. The TMEM30A^W260A^ expressing cells had similar annexin staining levels as wild-type cells indicating the cells had little exoplasmic PS (Fig 3A and 3B). However, these same cells had elevated levels of exoplasmic PE as indicated by duramycin staining (Fig 3A–3C). This contrast between exoplasmic PS and PE provided a unique opportunity to distinguish the lipid flipping activities of TMEM30a. Indeed, challenging these cell lines with MNV^CR6^ indicates that exoplasmic PS is not sufficient to explain why MNV requires lipid asymmetry. In particular, BV2ΔTMEM30a + TMEM30a^W260A^ cells have elevated exoplasmic PE and were unable to support MNV^CR6^ infection despite normal levels of exoplasmic PS (Fig 3D). Importantly, the TMEM30a complemented and deficient cell lines grew at similar rates, ruling out differences in viability as a basis for the differences in MNV replication (Fig 3E).These data suggest that the lipid flippase activity of TMEM30a is critical for promoting MNV replication and that MNV is more sensitive to exoplasmic PE rather than exoplasmic PS. Consistent with this model, masking the exoplasmic PS on BV2ΔTMEM30a cells by preincubating cells with saturating amounts of annexin V had no impact on viral binding to BV2 or BV2ΔTMEM30a cells (Fig 3F). Taken together, these data demonstrate that TMEM30a lipid flipping is critical for MNV recognition of cells and that this dependency on lipid asymmetry is independent of exoplasmic PS and correlates with exoplasmic PE.

Given that exoplasmic PS was not sufficient to explain MNV infection dependence on TMEM30a, we next explored whether a general disruption of lipid packing and order could be responsible for the phenotype. In addition to headgroup asymmetry, lipids are asymmetrical in lipid saturation which leads to distinct changes in membrane order and fluidity [37]. To characterize the physical properties of membranes we leveraged the outer leaflet selective probe NR12S. NR12S is a derivative of Nile Red whose fluorescence emission shifts towards shorter wavelengths (530 nm) when incorporated into a liquid-ordered phase compared to a liquid-disordered phase (630 nm) [38]. NR12S fluorescence emission profiles of BV2ΔTMEM30a cells demonstrated a significant increase in membrane disorder as measured by the emission ratio 530 nm/630 nm (Fig 3G). Importantly, this phenotype could be restored via complementation with a TMEM30a cDNA (Fig 3G). These data indicate that in addition to changes in lipid head group, TMEM30a is required to maintain exoplasmic lipid order, which has the potential to impact MNV binding and entry independent of lipid headgroup.

## Discussion

Here we uncover an unappreciated connection between lipid asymmetry and norovirus entry. Unlike enveloped viruses that disrupt lipid asymmetry to promote their uptake, MNV requires lipid asymmetry to enter cells [5]. These findings add to the growing body of literature that lipids are important mediators of norovirus entry. Sphingolipid biosynthesis maintains CD300lf in a functional confirmation for MNV entry [34]. However, CD300lf staining was not affected by disruptions in lipid asymmetry, pointing to a distinct role for the ordered distribution of lipids in promoting MNV infection (Fig 2C). For human norovirus, the addition of ceramide to cells promotes norovirus replication and cellular acid sphingomyelinase promotes a membrane wound repair pathway that enables entry of pandemic human norovirus strains [33,39]. However, neither the addition of C2 ceramide nor bile acids was able to rescue MNV entry in BV2ΔTMEM30a cells (Fig 2E). Thus, TMEM30a functions at an unappreciated stage of MNV binding that may involve lipid engagement with the viral receptor or the virion itself. Whether other noroviruses, including Human Norovirus requires TMEM30a is unknown and warrants investigation.

Noroviruses can be shed long after symptom resolution in asymptomatic individuals [9, 10]. In the MNV system, only a small number of tuft cells in the intestinal epithelium are infected despite high titers of virus being shed in the feces of the mice [25,40]. Disruption of lipid asymmetry in intestinal epithelial cells dramatically reduces viral persistence in vivo (Fig 1K and 1L). Given that TMEM30a deficient cells can support low levels of viral replication in vitro, these data suggest that only a small perturbation to persistent viral infection may be necessary to halt transmission cycles. One limitation of our study is that we are unable to ascertain whether defective MNV entry is the reason why *TMEM30a*^*fl/fl*^*-Vilcre* mice do not support persistent infection of MNV^CR6^ or whether other complex non-cell autonomous factors are responsible. Nevertheless, TMEM30a is required for persistent MNV infection and represents a potential therapeutic target for persistent norovirus infections.

Lipid asymmetry involves the segregation of different lipid classes to distinct leaflets of the lipid bilayer. Disruption of lipid asymmetry in platelets leads to a ratio of two PSs for every five PEs being exposed [41]. Despite the abundance of exoplasmic PE, most research efforts have focused on the distribution of PS given the number of scavenger receptors that bind PS to facilitate the clearance of dead cells [42]. Exoplasmic PE has been shown to have a similar role for PS or synergize with PS engagement of proteins [43, 44]. In contrast, our work correlates a role for exoplasmic PE in preventing MNV entry. Using a TMEM30a mutant that has a partial loss of function (TMEM30A^W260A^), we generated cells that had exoplasmic PE but normal PS distribution (Fig 3A and 3B). In this setting, MNV was unable to replicate, suggesting a critical role of PE but not PS in regulating MNV entry (Fig 3D). How PE directly or indirectly impacts viral binding to cells is an outstanding question. We propose a model in which changes in the distribution of lipids alter membrane fluidity which in turn reduces MNV binding. This model is supported by the recent finding that decreasing membrane mobility reduced MNV binding due to the low-affinity, high avidity of MNV engaging CD300lf [32,45]. Indeed, in our in vitro model, BV2ΔTMEM30a cells had a decrease in lipid order, one component that influences membrane fluidity (Fig 3G).

However, several alternative, non-exclusive models should be noted. First, the organization of lipid microdomains is an important aspect of cellular signaling. While signaling from CD300lf itself is not required for MNV entry, it is possible that non-CD300lf signaling events that lead to the clustering or stabilization of MNV-CD300lf complex are missing in BV2ΔTMEM30a cells [15]. Additionally, the distribution of lipids is important for endomembrane trafficking events. Thus, it is possible that the disruption of lipid asymmetry impacts the subcellular localization and trafficking of both transmembrane and peripheral membrane proteins. While CD300lf itself is trafficked to the cell surface efficiently in both BV2ΔTMEM30a and BV2 wild-type cells (Fig 2C), other unappreciated MNV entry proteins may be mislocalized in TMEM30a deficient cells. Finally, many viruses bind lipids or glycolipids to enhance their entry into host cells [46, 47]. We have been unable to demonstrate a role in MNV entry for cellular lipids or glycans directly engaging the MNV capsid [15]. However, it is possible that a complex, cooperative biochemical interaction exists between lipids, CD300lf, and the MNV capsid that is disrupted in TMEM30a deficient cells. Future work leveraging advances in viral genetics, cell biology, and lipid biochemistry are needed to further define the interesting but complex role of TMEM30a in MNV entry.

Our work has several limitations. First, the mechanism for how the TMEM30a^W260A^ mutant selectively enables PS flipping but not PE is unknown. This phenotype may be cell-type specific or dependent upon the expression levels we achieved in our study. Second, we are currently unable to disentangle lipid saturation asymmetry and head group asymmetry. It is possible that changes in both lipid packing and lipid headgroups are detrimental to MNV infection. Despite over 50 years of research on membrane biology, it remains unclear why an asymmetric bilayer is advantageous to cells [37,48]. Our research results presented here provide new tools and a physiological system to address this fundamental aspect of cellular physiology.

## Materials and methods

### Ethics statement

All mouse experiments were conducted at University of Texas Southwestern Medical Center and approved by the University of Texas Southwestern Medical Center’s Institutional Animal Care and Use committees under protocol 2018-102627.

### Cell culture

293T (ATCC), Vero (ATCC) and the mouse microglial cell line BV2 (Kind gift of Dr. Skip Virgin, Washington University) were cultured in Dulbecco’s Modified Eagle Medium (DMEM) with 5% fetal bovine serum (FBS) [49]. 3 µg/mL of puromycin (Thermo Fisher) was added as appropriate. BV2ΔCD300lf cells have been described previously [15]. BV2ΔTMEM30a cells were generated at the Genome Engineering and iPSC center at Washington University School of Medicine. BV2 cells were nucleofected with Cas9 and TMEM30a-specific sgRNa (CGGAGATAAACATTTATTGCNGG). Single cell clones were screened for frameshifts by sequencing the target region with Illumina MiSeq at approximately 500× coverage. All cell lines are tested regularly and verified to be free of mycoplasma contamination.

### Plasmids

cDNA for human TMEM30a was obtained from TransOMIC and subsequently cloned into pCDH-MSCV-T2A-Puro vector (System Biosciences) with and without a C-terminal HA-tag. pCDH-MSCV-CD300lf-Flag-T2A-Puro was described previously [15]. All TMEM30a mutants were generated through splicing by overlap extension PCR. All DNA constructs were sequence verified.

### VSV experiment

5 x 10^4^ wild-type BV2 or BV2ΔTMEM30a cells were seeded in a 96-well plate overnight. The following day, media was removed and VSV^Indiana^ (a kind gift of Sean Whelan) was added to cells at an MOI of 0.5. After 1 h of gentle rocking at room temperature, viral inoculum was removed, washed with phosphate-buffered saline (PBS), and fresh media to the cells. Sixteen hours post-infection samples infections were quantified using a standard plaque assay with Vero cells.

### MNV assays

MNV^CW3^ (Gen bank accession no. EF014462.1), MNV^CR6^ (Gen bank accession no. JQ237823), and MNV^CR6^-HiBiT were generated by transfecting molecular clones into 293T cells and amplifying on BV2 cells as described previously [15,35]. MNV strains CR3, CR7, WU23, and S99 were kind gifts from Dr. Craig Wilen (Yale School of Medicine) [26]. For MNV growth assays, 5 × 10^4^ BV2 cells were seeded in 96-well plates and the next day inoculated with virus and frozen at −80 °C at the indicated time point. MNV plaque and entry bypass assays were done as described previously [15]. For isolation of MNV^CR6^ RNA, RNA was purified from cell-free viral preparations using the Direct-zol RNA Miniprep kit (Zymo Research catalog #R2052) according to manufacturer instructions. Purified RNA, which includes both host and viral RNA, was verified to be free of infectious MNV particles via plaque assay. Ten µg of RNA was transfected using Lipofectamine 2000 transfection reagent according to the manufacturer’s protocol. Transfected cells were frozen 12 h later. All infections were done in triplicate in each of at least three independent experiments.

MNV^CR6^ binding assays were performed as previously described [34]. Briefly, MNV^CR6^ binding to BV2 cells was performed for 1 h at 4 °C in 0.5 mL DMEM with 10% FBS. BV2 cells were used at a final concentration of 2.5 × 10^6^ cells/mL and MNV was used at an MOI of 1. Cells were centrifuged 500*g* for 5 min at 4 °C to remove unbound virus. Cells were washed four times with 1 mL cold DMEM with 10%FBS. The cell pellet was resuspended in 100 µ L PBS, and RNA was extracted using the Direct-zol RNA Miniprep kit according to manufacturer instructions. qPCR was utilized to quantify MNV and Actin transcript abundance. Binding assays were conducted in triplicate in at least three independent experiments and binding was normalized within an experiment to the ratio of MNV/Actin and normalized between experiments by setting the relative binding of MNV to WT BV2 cells at 100% as done previously [34].

For MNV^CR6^-HiBiT experiments, 2.5 × 10^5^ BV2 or BV2ΔTMEM30a cells were seeded in a 24-well plate overnight. The next day media was replaced with DMSO, 500 µ M GCDCA (Sigma), or 50 µ M C2 ceramide (Avanti Research) with 1.25 × 10^5^ PFU of MNV^CR6^-HiBiT in 200 µ L total volume. After 1 h of incubation at room temperature with gentle rocking, media was removed and 200 µl of standard media was replaced (DMEM with 5% FBS). After 16 h, cells were lysed in 200 µl of Nano-Glo HiBiT Lytic Buffer (Promega). Lysed samples were then mixed LgBiT protein and Nano-Glo HiBiT Lytic Substrate prior to reading luciferase as detailed in manufacturer’s protocol. Each experimental condition was performed in triplicate across three independent experiments. Within each experiment samples were normalized to the average signal of the wild-type BV2 cells treated with DMSO.

### Cell survival assays

For cell survival assays, 2 × 10^4^ of indicated cell lines were seeded in wells of a white-walled 96-well plate (Corning) either untreated (mock) or with MNV^CR6^ at indicated MOI. Twenty-four or 48 hlater, viability was measured using CellTiter-Glo (Promega) according to manufacturer’s protocol. Within each experiment, the viability of each cell line was normalized to the untreated of the wild-type BV2 (or wild-type BV2 expressing an empty vector). Each condition was assessed at least in quadruplicate in three independent experiments.

### Flow cytometry

For fluorescence-activated cell sorting (FACS) analysis, cells were isolated and probed for either Annexin-V (Biolegend #64095) or Duramycin-LC-Biotin (polysciences catalog#25690-100) in annexin staining buffer (0.01 M Hepes Buffer, 0.14 M NaCl, 2.5 mM CaCl_2_) or PBS respectively. Samples were incubated at 4 °C for 30 min prior to washing and staining with FITC- or PE- conjugated Streptavidin. The fluorescent intercalator 7-Aminoactinomycin D (7-AAD) (Fisher Scientific catalog#A1310) was used as a control to observe cell-viability. For CD300lf expression, cells were stained with the PE-TX70 antibody (Biolegend #132704) as described previously[34]. For intracellular FACS, cells were fixed and permeabilized with Cytofix/Cytoperm (BD Biosciences) prior to staining Rabbit monoclonal anti-HA antibody (CellSignaling Technologies #3724). At least 20,000 events were collected per condition. Each experiment was performed in triplicate in each of three independent experiments. Cells were analyzed on a FACS Calibur flow cytometer and data analyzed using FlowJo v10 (FlowJo LLC, Ashland, OR).

### Mouse infections

We thank the Wellcome Trust Sanger Institute Mouse Genetics Project (Sanger MGP) and its funders for providing the mutant mouse line *Tmem30atm1a(KOMP)Wtsi* and INFRAFRONTIER/EMMA (www.infrafrontier.eu). Funding information may be found at www.sanger.ac.uk/mouseportal and associated primary phenotypic information at www.mousephenotype.org. *Tmem30atm1a(KOMP)Wtsi* mice were first bred to *B6.Cg-Tg(Pgk1-flpo)10Sykr/J* (Jax stock #011065) to remove the genetrap cassette and create a conditional knockout mouse. We then bred *TMEM30a*^*fl/fl*^ mice to *B6 Vil-cre* (Jax stock #004586) in a pathogen-free barrier facility including devoid of murine norovirus. 1 × 10^6^ PFU of MNV^CR6^ diluted in 25 µl of DMEM was inoculated per orally to 6–8-week-old mice. Immediately after inoculation, mice were singly housed. Fecal pellets were collected at 0, 3, 7, 14, and 21 days post-infection. Mice were euthanized at 21 days post-infection and tissues were harvested and prepared for RNA isolation.

### Quantitative PCR

MNV genome copies in fecal pellets and tissues were determined as previously described. Briefly, RNA was isolated from infected tissues using TRI Reagent (Sigma- Aldrich # T9424) with a Direct-zol kit (Zymo Research) following the manufacturers’ protocols. 1 µg of RNA was used for cDNA synthesis using a High-Capacity cDNA Reverse Transcription kit, following the manufacturer’s protocols (Thermo Fisher Scientific # 4368813). TaqMan quantitative PCR (qPCR) for MNV was performed in triplicate on each sample and standard with forward primer 5′-GTGCGCAACACAGAGAAACG-3′, reverse primer 5′-CGGGCTGAGCTTCCTGC-3′, and probe 59-6FAM-CTAGTGTCTCCTTTGGAGCACCTA-BHQ1-3′. TaqMan qPCR for Actin was performed in triplicate on each sample and standard with forward primer 5′-GATTACTGCTCTGGCTCCTAG-3′, reverse primer 5′-GACTCATCGTACTCCTGCTTG-3′, and probe 5′-6FAM-CTGGCCTCACTGTCCACCTTCC-6TAMSp-3′. RNA from infected fecal pellets was isolated using RNeasy Mini QIAcube Kit (Qiagen # 74116) and cDNA synthesis was performed using M-MLV Reverse Transcriptase kit (Invitrogen # 28025013) using manufacturer’s protocols.

### NR12S

5 × 10^4^ BV2 cells were seeded in black-walled 96-well plates and the following day media was removed and 100 nM NR12S (Tocris) diluted in DMEM without FBS was added to cells at room temperature for 20 min. Media was removed, and PBS was added to cells. Samples were read on a BioTek Cytation5 plate reader with an excitation of 520 nm and separate emission readings at 560 and 630 nm.

## Supporting information

S1 Table Primary data for all figures. (XLSX)

## Abbreviations

FACS: fluorescence-activated cell sorting
GCDCA: glycochenodeoxycholic acid
MNV: Murine norovirus
MOI: multiplicity of infection
NoV: Noroviruses
PBS: phosphate-buffered saline
PE: phosphatidylethanolamine
PS: phosphatidylserine
VSV: vesicular stomatitis virus
